# Anæmia with Amenorrhœa

**Published:** 1890-05

**Authors:** 


ANÆMIA WITH AMENORRHŒA.—J. Milner Fothergill's prescription for amenorrhœa accompanied by anæmia, is as follows:

| | | |
|---|---|---|
| ℞. | Acidi arseniosi - - - - - | gr. j. |
| | Ferri sulphat. exsiccat - - - - | ʒss. |
| | Pulv. pip. nigr. - - - - - | ʒj. |
| | Pil. aloes et myrrhæ - - - - | ʒj. |

M. Et div. in pil. No. xl.

Sig. One twice a day, after meals.

